# 9-year follow-up of uncommon cleft palate in Aarskog-Scott syndrome

**DOI:** 10.4317/jced.60560

**Published:** 2023-07-01

**Authors:** Andreza-Maria-Fábio Aranha, Kadyja-de Assis Veiga, Maria-Angélica-Liesenberg Stefanini, Yolanda-Benedita-Abadia-Martins de Barros, Orlando-Aguirre Guedes

**Affiliations:** 1 DDS, MSc, PhD. Department of Dental Sciences, School of Dentistry, University of Cuiabá, Cuiabá, Brazil; 2 DDS, MSc. Department of Dental Sciences, School of Dentistry, University of Cuiabá, Cuiabá, Brazil; 3 DDS, MSc, PhD. Department of Oral Sciences, School of Dentistry, Evangelical University of Goiás, Anápolis, Brazil

## Abstract

Aarskog-Scott syndrome (AAS) is characterized by different facial, skeletal and genital anomalies and may have oral manifestations. A 7-year-old boy was referred to the University General Hospital for treatment of speech difficulties and frequent regurgitation. Characteristics such as a triangle-shaped face, hypertelorism, low-set ears, flattened nose, shawl scrotum and partial syndactylia on hands and feet were observed. Based on these clinical features, the child was diagnosed with AAS. Upon intraoral examination, maxillary atresia and an incomplete cleft palate were observed. The mixed dentition was characterized by extensive coronary destruction of primary teeth and caries lesions on permanent teeth. Here, the case of a 9-year follow-up of this child with uncommon AAS associated with cleft palate is reported. The child was referred to a multidisciplinary team for planning and carrying out the treatment. In the follow-up visit after 9 years from the beginning of the treatment, the child showed greater sociability, with significant improvement in spontaneous speech and pronunciation of phonemes. However, the patient continues until now with articulation and spontaneous speech training. The correction of class II malocclusion, better dental alignment and canine extrusion were achieved. At the moment, the patient uses a nighttime extraoral device, and the treatment continues for dental alignment and prevention of tooth decay. The presence of cleft palate could be coincidental with AAS and may aggravate the prognosis, requiring careful patient monitoring by a multiprofessional team.

** Key words:**Aarskog-Scott syndrome, cleft lip, cleft palate, multidisciplinary treatment.

## Introduction

*Aa*rskog-Scott syndrome (AAS) comprises a rare X-linked recessive entity caused by mutation in the FGD1 gene mapped to the Xp11.21 region (1) and is characterized by distinctive facial, skeletal and genital developmental abnormalities (2). Oral manifestations have also been described as part of the clinical characteristic of AAS (3) in particular, both maxillary and mandibular hypoplasia (2), retardment in the development and eruption of permanent teeth, hypodontia, taurodontism, enamel dysplasia, orthodontic anomalies and high prevalence of dental caries (4-6). Since AAS is a genetically variable disease, a considerable spectrum of clinical phenotypes has been reported, making the clinical diagnosis difficult (3,7-9).

Cleft lip and palate (CLP) are among the most common congenital craniofacial defects, and although their etiology has not been clearly established, there is evidence that genetic and environmental factors are directly related (10). Individuals with CLP may face problems with eating, speaking, listening and social integration, requiring an interdisciplinary approach to the treatment of skeletal and dental changes (10). Changes in the morphology of the jaws may result in changes in the arch such as the agenesis of lateral incisors, supernumerary teeth, crowding, abnormalities in tooth form, hypodontia, changes in the chronology of dental eruption, maxillary atresia and, posterior crossbite (11). The earlier the diagnosis and treatment of congenital deformities, the better the acceptance of the family unit and society, and the better the prognosis for the case (10). Children with cleft lip and/or palate may have associated malformations, whether or not they are included in a syndromic form, with cleft lip and labioalveolar clefts being less frequently associated with other malformations than cleft palate (11). The present article describes the clinical case of a 9-year follow-up of an uncommon association between AAS and cleft palate.

## Case Report

**Figure 1:**
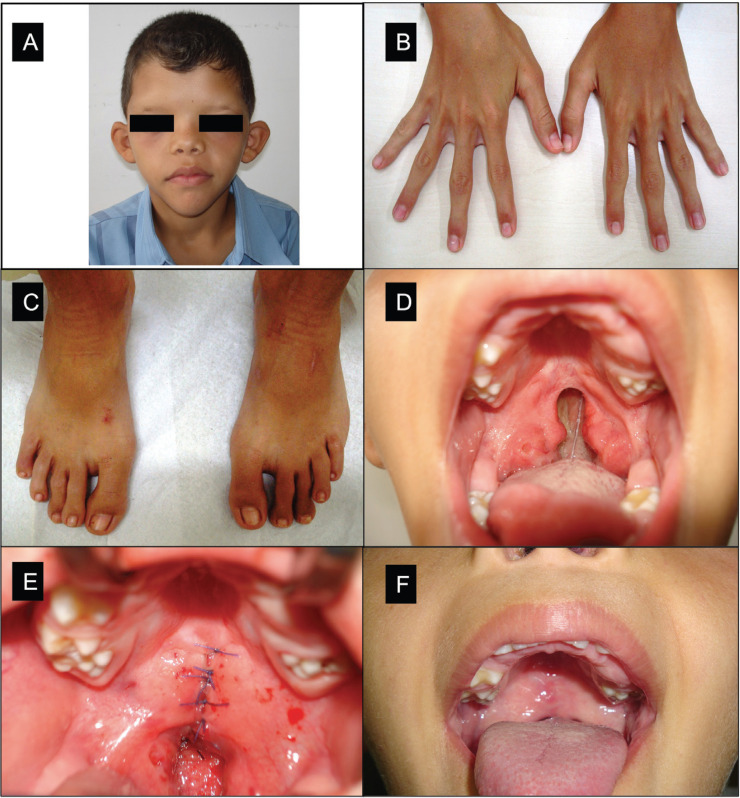
Physical and oral features of Aarskog-Scott syndrome and cleft palate. (A) Extraoral photo showing hypertelorism, low-set ears, flattened nose and triangle-shaped face; (B–C) Broad hands and feet with mild interdigital webbing; (D) Maxillary atresia and incomplete cleft palate; (E) Palate closure immediately after the surgery; (F) 30 days after the palatoplasty.

In November 2006, a 7-year-old Caucasian boy was referred to the University General Hospital for the chief complaint of speech difficulties and frequent regurgitation. Upon physical examination, characteristics such as a symmetrically triangular face, hypertelorism, apparently low-set ears, flattened nose tip, shawl scrotum and partial syndactylia on hands and feet were observed (Fig. 1A-C). Based on these clinical features, the child was diagnosed with Aarskog-Scott syndrome. Upon interview, the mother reported this had been her first pregnancy and that she had been received prenatal care, with the absence of any complications. She denied the use of alcohol, drugs or medication during pregnancy until normal child delivery.

Upon intraoral examination maxillary atresia and an incomplete cleft palate (Fig. 1D) were observed that had previously been diagnosed but the mother had not sought medical attention. The mixed dentition was characterized by extensive coronary destruction of primary teeth including the first and second right upper molars (#55, #54), upper right canine (#53), right mandibular lateral incisor and canine (#72, #73) and left mandibular canine and first molar (#83, #84) and caries lesions in the first permanent molars (#26, #36 and #46) and an active white spot on the occlusal surface of the upper right first permanent molar (#16).

The boy underwent further laboratory tests, which diagnosed him with hematuria and an abnormal number of red blood cells being deleted in the urine. During the speech evaluation, the mother reported a history of breastfeeding, regurgitation of food through the nose during swallowing, ear and throat infections until 3 years old and speech difficulties. Stroke of the glottis, omissions and exchanges of phonemes, and the presence of hypernasality due to changes in the velopharyngeal sphincter were observed. A hearing impairment with mild sensorineural hearing loss in the right ear and moderate sensorineural loss in the left ear were also diagnosed, with impaired hearing acuity and discrimination of sounds.

Finally, the patient treatment planning was carried out by a multidisciplinary team in the Rehabilitation Service of Cleft Lip Palate at the hospital, which determined the following sequence of treatment: dental care (rehabilitation of the oral environment by dental extractions, occlusal sealing of tooth #16 and aesthetic restorations), psychological and speech evaluation (use of an individual sound amplification device—ISAD), urology, and subsequent palate reconstruction (partial palatoplasty) and orthodontic treatment.

In February 2009, the child underwent surgical closure of the soft palate (palatoplasty) using the von Langenbeck technique. Briefly, this procedure consists of repairing both the hard and soft palates, tension-free approximating the mucoperiosteal and soft palate flaps, and everting suture of the nasal and oral mucous membranes from the anterior extent of the palate cleft to the tip of the uvula. The palatoplasty was performed under general anesthesia and infiltration anesthesia of the hard and soft palates (lidocaine 2% with adrenaline 1:200,000) (Dentsply Maillefer, Petrópolis, RJ, Brazil) (Fig. 1E). Postoperatively 30 days from surgery the patient showed good cicatricial clinical condition and progress in speech therapy (Fig. 1F).

**Figure 2:**
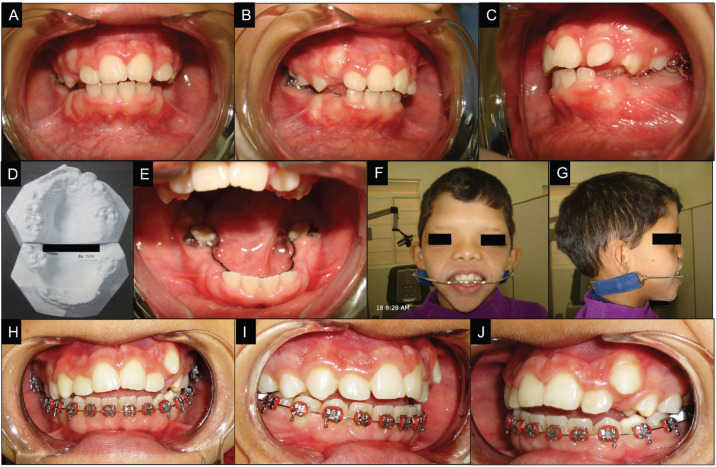
Diagnosis and treatment of angle class II malocclusion. (A) Frontal view; (B) Right side view; (C) Left side view; (D) Diagnostic study models; (E) Nance lingual arch for maintaining the space in the arch; (F–G) Extraoral device for distal movement of the upper molars; (H–J) Correction of dental alignment.

After palatoplasty, in August 2010, the patient was referred back to dentistry for orthodontic treatment to correct Angle class II malocclusion, a lack of space in the maxillary arch with interference in the eruption of the canines. Orthodontic planning consisted of the installation of a Nance lingual arch to preserve space after early loss of deciduous lower molars, an extraoral device for distal movement of the upper molars, correction of dental alignment and extrusion of the upper left canine (Fig. 2A-J).

At a follow-up appointment 9 years after the beginning of the treatment, new laboratory tests showed no urological disorders. The child showed greater sociability, with significant improvement in spontaneous speech and pronunciation of phonemes (L, R and S). However, he still had difficulties pronouncing the phonemes F and V. Thus, the patient continues until now with articulation and spontaneous speech training. Due to the noncooperation of the child with respect to performing dental hygiene, orthodontic treatment had to be stopped several times. Still, it was possible to correct the class II malocclusion, improve dental alignment and extract the canine in question. At this time, the patient nocturnally uses the extraoral device and continues with dental alignment treatment and a dental decay prevention program.

## Discussion

AAS comprises facial, digital and genital anomalies, and although great attention has been given to the systemic alterations associated with this syndrome, limited information is available regarding the oral conditions (5). Here, the case of a 9-year follow-up of a child with uncommon AAS associated with cleft palate was reported.

The diagnosis of AAS is made primarily on the basis of clinical criteria. In typical cases, short stature, distinct craniofacial abnormalities, cardiac defects and skeletal anomalies (scoliosis, brachydactyly, broad hands and interdigital webbing) are observed. In addition, the phenotype of affected males is characterized by genital anomalies, such as shawl scrotum, cryptorchidism and inguinal hernia (2,4,6,8,9). Although molecular diagnostic methods were not undertaken, the present case satisfied most of the characteristics of the syndrome, such as hypertelorism, shawl scrotum, partial syndactylia on hands and feet, triangular face, changes in auricular pavilion and maxilla hypoplasia. Orrico *et al*. (8) proposed that the presence of relatively few signs (i.e., the association of three or more classical signs) might be sufficient for the clinical suspicion of AAS. Nevertheless, individuals with AAS can present a variety of clinical features, which can hinder the diagnosis and require the differential diagnosis with other syndromes (3,8,9). The differential diagnosis of AAS includes Noonan, Robinow and SHORT syndromes as well as pseudohypoparathyroidism (2,7,8). Teeby *et al*. (7) proposed primary and secondary diagnostic criteria for AAS. The adoption of these criteria has allowed the correct differentiation between AAS and other syndromes.

Many oral conditions have been associated with AAS: retarded development and eruption of the permanent teeth, maxilla, and mandible hypoplasia, negative dentoalveolar discrepancy, micrognathia, hypodontia, orthodontic anomalies and high prevalence of dental caries (5,6,9). The presence of cleft lip and cleft palate in patients with AAS has been previously reported; however, the existence of a correlation between these two entities has not been well established (1,4,12). For Furukawa *et al*. (4), AAS does not appear to have cleft lip or palate association. In contrast, de Saxe *et al*. (12), after examining six affected males from three families and observing the presence of cleft lip in one patient, suggested that both cleft lip and/or cleft palate may well be an oral feature of AAS. De Wolf *et al*. (1) presented the case of a male patient with features of AAS, intellectual disability, autism and cleft palate. The distribution of malformations appears to be linked to the type of cleft. However, cleft palate appeared to be much more frequently associated with a higher rate of other malformations or syndromes (11). Thus, the presence of cleft palate could be coincidental with AAS.

AAS is caused by mutations in the FGD1 gene located in the Xp11.21 region (1,8,9). The FGD1 gene encodes a guanine nucleotide exchange factor (GEF), which activates Cdc42 and the c-Jun N-terminal kinase, important signaling pathways involved in cytoskeletal organization, skeletal formation and morphogenesis (3). Since cleft palate is not a common oral manifestation of AAS (4), the deletion of FGD1 does not explain its presence. The presence of cleft palate may be explained by the deletion of the PHF8 gene, also located in the Xp11.21 region (1,13-15). PHF8 presents two functional domains (PHD finger and a JmjC domain) and plays a critical role in transcriptional regulation and chromatin remodeling (13). It has been reported that chromatin-regulated gene expressions are commonly associated with congenital abnormalities (15). Recently, the PHF8 gene has been found to cause Siderius X-linked mental retardation syndrome, whose symptoms include mild mental retardation, facial dysmorphism and cleft lip/palate (14).

In the present case, the combination of AAS and cleft palate resulted in maxillary atresia, severe dental crowding and early tooth loss. As there should be no differences in the orthodontic treatment of individuals with or without the syndrome and cleft palate (6), the treatment of the present patient consisted of obtaining and preserving space in the dental arch (through a Nance lingual arch and an extraoral device) and, later, of dental alignment (braces). Although regular fluoride use, oral care guidelines and dietary recommendations were implemented during the follow-up visits, the treatment lasted for a longer period than normal due to poor patient compliance with respect to oral hygiene, resulting in interruption several times.

Since pathognomonic signs appear when individuals with AAS are aged between 2 and 4 years (6), a preventive approach is the key to monitor and intercept the development of caries lesions and consequent early tooth loss. A well-informed and attentive clinician or an effective interdisciplinary team will contribute to the earlier diagnosis and appropriate treatment of these patients.
